# Implementation of a Parent Training Program During Community-Based Dissemination (From In-Person to Hybrid): Mixed Methods Evaluation

**DOI:** 10.2196/55280

**Published:** 2024-07-03

**Authors:** Heather McGrane Minton, Linda Murray, Marjorie J Allan, Roslyn Perry, Amie F Bettencourt, Deborah Gross, Lauri Strano, Susan M Breitenstein

**Affiliations:** 1 Wegmans School of Nursing St. John Fisher University Rochester, NY United States; 2 Children's Institute University of Rochester Rochester, NY United States; 3 College of Nursing The Ohio State University Columbus, OH United States; 4 School of Medicine Johns Hopkins University Baltimore, MD United States; 5 School of Nursing Johns Hopkins University Baltimore, MD United States

**Keywords:** COVID-19, implementation, internet-based intervention, parenting, community dissemination, hybrid delivery

## Abstract

**Background:**

Parent training interventions support and strengthen parenting practices and parent-child relationships and improve child behavior. Between March 2018 and February 2020, a community-based parenting program conducted 38 in-person Chicago Parent Program (CPP) groups. In response to the COVID-19 pandemic, we modified the delivery of the in-person CPP to hybrid delivery using the self-administered, web-based version of the CPP (*ez*Parent) paired with web-based, videoconferenced group sessions.

**Objective:**

This study aims to describe the delivery transition and implementation outcomes of the hybrid delivery of the CPP (ezParent+group) during community-based dissemination.

**Methods:**

This single-group, mixed methods retrospective evaluation examined the implementation outcomes using the RE-AIM (Reach, Efficacy, Adoption, Implementation, and Maintenance) framework. We report on data from hybrid *ez*Parent delivery between September 2020 and August 2022. Parents completed pre- and postprogram surveys that included motivation to participate and perceived changes in parent-child behavior. Digital analytics captured *ez*Parent completion. Facilitators completed fidelity assessments and participated in postintervention interviews.

**Results:**

In total, 24 hybrid *ez*Parent groups (n=240 parents) were delivered by 13 CPP-trained facilitators. Parents reported high levels of satisfaction with the program and improvements in their feelings of parenting self-efficacy and their child’s behavior following their participation in hybrid *ez*Parent. On average, parents completed 4.58 (SD 2.43) 6 *ez*Parent modules. The average group attendance across the 4 sessions was 71.2%. Facilitators found the hybrid delivery easy to implement and reported high parent engagement and understanding of CPP strategies.

**Conclusions:**

Using the hybrid *ez*Parent intervention is a feasible and effective way to engage parents. Lessons learned included the importance of academic and community-based organization partnerships for delivering and evaluating robust programs. Implementation facilitators and barriers and future research recommendations are discussed.

## Introduction

### Background

Behavior parent training (PT) is considered the gold standard for supporting and strengthening parenting practices and parent-child relationships and for the prevention and treatment of child behavior problems [1,2]. Positive parenting practices are a protective factor in buffering the negative effects of childhood trauma or early adverse experiences [3,4]. Parenting practices are often informed by family of origin, community, and social connections, and parents often look to their community for parenting support and guidance and many community-based agencies offer evidence-based PT [3].

Unfortunately, community organizations delivering PT experienced major disruptions in the provision of services with an abrupt ending of all in-person services due to the onset of the COVID-19 pandemic and shelter-in-place orders. These disruptions exacerbated an existing access gap for many communities, particularly those who were providing in-person individual or group-based PT. In addition, while parents lost access to parenting supports and child activities (eg, group activities, child care, school, and playgrounds), they experienced a substantial burden of shelter-in-place requiring a balance of work and parenting responsibilities [5,6]. Partially due to social isolation, balancing of responsibilities, and limited access to information and resources, data suggest a surge in negative mental, emotional, and physical health outcomes for parents and children [5,7,8]. Therefore, resources and support to mitigate the effects of the pandemic were crucial during this time.

Many community organizations shifted delivery of in-person PT and other parent supportive services using technology (eg, using videoconferencing for groups and web-based programming) [9-12]. The purpose of this paper is to describe the transition of a group-based PT program (ie, the Chicago Parent Program [CPP]) to a hybrid delivery model using the web-based PT of the CPP (*ez*Parent) paired with videoconferenced group sessions and implementation in a community-based parent support program. This study is the result of a community-academic partnership in which representatives from each coled the design, implementation, and interpretation of the data.

### The Chicago Parent Program

The CPP is for parents of children aged 2 to 8 years and designed as a strength-based approach to promote positive parenting skills (ie, warmth, positive engagement, and support), support optimal child social and emotional development, and enhance parent-child interactions [13-16]. The CPP is a 12-session, group-based program that was developed with input from an advisory board of Black and Hispanic parents in low-income neighborhoods and aims to be contextually and culturally relevant for families from diverse racial, ethnic, and income backgrounds. Currently, the third edition of the CPP is broadly used in community agencies, pediatric primary care, early childhood care and education centers, and schools [17].

*ez*Parent is the web-based version of the CPP and was designed to deliver the core parenting skills and strategies in the CPP via 6 self-directed modules. Similar to the group-based delivery, *ez*Parent content and delivery was informed by an advisory group of Black and Hispanic parents and designed to help parents develop positive and effective parenting skills and decrease physical punishment through the use of behavioral strategies (eg, routines and labeled praise), brief videos of parents using the strategies, activities, quizzes, and practice assignments [18]. The web-based delivery of the CPP was developed to increase access by addressing challenges for parents and providers related to in-person delivery, including logistic barriers (eg, work demands, scheduling, childcare, and transportation), access to in-person groups, and potential for stigma [18,19].

### Rochester Area Parenting Program

The Rochester Area Parenting Program (RAPP), a program initiative at the Children’s Institute Inc in Rochester, New York, began implementing the group-based CPP in 2018. From March 2018 through March 2020, RAPP supported 35 CPP groups in collaboration with 6 partnering community programs across 14 sites (eg, churches, Head Start, childcare centers, and a large urban school district). During this time, 396 parents participated in CPP groups led by 23 CPP-trained group leaders. A key community engagement parent empowerment and leadership initiative of RAPP is to identify parents who completed CPP groups and subsequently support these parents in completing CPP training to become group leaders; thus, 25% of CPP-trained group leaders were parent graduates of CPP groups. At the onset of the COVID-19 pandemic shelter-in-place orders (ie, March 2020), RAPP suspended 3 in-person CPP groups and canceled 2 groups that were scheduled to start and began exploring options for alternative delivery methods of CPP to assure continued service provision and support for the families they serve. The RAPP coordinators engaged the CPP developers to determine options for delivery. During this time, many in-person CPP groups nationally moved to web-based delivery using videoconferencing platforms and adapted aspects of group delivery (eg, role-play, group activities, and discussion format). This option was presented to the RAPP leadership team, a group that provides strategic leadership and input on RAPP programming. The RAPP leadership team is a community collaboration with RAPP administrators and agency directors, staff, parent graduates of RAPP programming, trained facilitators, and faculty experts from partnering community agencies, schools, and universities. The RAPP leadership team reached consensus that 2-hour videoconferenced groups over 12 weeks would be challenging for the parents they serve due to logistics and competing demands on parents’ time. In addition, the RAPP leadership team considered parent feedback from the group-based CPP indicating that parents highly valued peer and facilitator interaction and support. Therefore, RAPP worked closely with the CPP developers to develop a hybrid model of delivery where parents would complete *ez*Parent modules independently and participate in four 1-hour videoconferenced group sessions led by 1 CPP-trained facilitator.

The purpose of this paper is to describe the implementation outcomes of the hybrid delivery model of self-administered, web-based PT (ie, ezParent) and group sessions during community-based dissemination. Specifically, we seek to evaluate the implementation outcomes, using the RE-AIM (Reach, Efficacy, Adoption, Implementation, and Maintenance) model, of delivering the *ez*Parent program, and identify facilitators and barriers to implementation delivery to support the sustainability of program implementation.

### RE-AIM Framework

The RE-AIM framework was designed to provide a model to enhance the quality, speed, and public health impact of efforts to translate research into practice using 5 dimensions: *reach* the intended target population; *efficacy* of the intervention and implementation strategies; *adoption* by trained facilitators and settings (agencies); *implementation*, including fidelity and consistency of delivery; and *maintenance* of intervention effects in individuals and implementation in settings over time [20,21]. In this evaluation, we will report on the reach, efficacy, adoption, and implementation components and present maintenance as part of the discussion.

## Methods

### Study Design

This single-group, mixed methods evaluation examined the implementation outcomes using RE-AIM of 24 hybrid *ez*Parent groups (n=240 parents) delivered by 13 CPP-trained facilitators in the northeastern United States. We report on survey and interview data from hybrid *ez*Parent delivery between September 1, 2020, and August 1, 2022.

### Ethical Considerations

Exempt approval for this project was granted by the Ohio State University Institutional Review Board (number 2022E0128).

### ezParent Hybrid Delivery

Although the change in delivery was reactive to the COVID-19 pandemic shutdown, the delivery modification was a thoughtful and engaged process occurring between April and August 2020. Modifications occurred as part of a collaborative effort between CPP and *ez*Parent developers, RAPP coordinators, and the RAPP leadership team to assure congruence with underlying CPP theory and content and RAPP family needs with the goal of maximizing intervention fit and implementation success. Refer to Textbox 1 for a description of key team members involved in the implementation of hybrid *ez*Parent. We describe the adaptation process in Table 1 using the last 4 of 8 categories of the Framework for Reporting Adaptations and Modifications–Expanded (FRAME) outline [22]. The FRAME provides a structure for reporting the delivery adaptation. The FRAME categories include what is changed or modified, at what level of delivery the modification is made, type or nature of context or content-level modifications, extent to which the modification is fidelity consistent, and the reasons for the modification (ie, the intent or goal of the change and contextual factors that influenced the decision). What did not change as part of the adaptation was the *ez*Parent program and core CPP skills and strategies, participation incentives provided for parents from RAPP, and groups facilitated by trained CPP group leaders.

**Figure 1:**
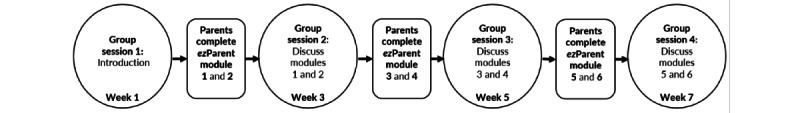
Hybrid *ez*Parent schedule of delivery.

The hybrid *ez*Parent program began with an introductory videoconferenced group session with the facilitator and parents. The purpose of this session was to introduce parents to the purpose of the *ez*Parent program and the groups sessions (eg, to review content parents were learning, review key strategies, and discuss parents’ successes and challenges in using the strategies) and to prepare them for the next session. The purpose of subsequent group sessions was to clarify the content, promote social connection and engagement, and help keep parents engaged in using *ez*Parent. At the introductory session, parents were instructed to complete module 1 and 2 over the next 2 weeks before the next group session. This schedule is repeated for modules 3 to 4 and 5 to 6, each followed by a group session (Figure 1).

**Key team members implementing hybrid ezParent.**

Title and role in hybrid *ez*Parent implementation

- **Chicago Parent Program (CPP) and *ez*Parent developers**: authors of CPP and ezParent
- **Facilitators**: CPP-trained *ez*Parent group facilitators
- **Parents**: hybrid *ez*Parent participants
- **Rochester Area Parenting Program (RAPP) coordinators**: administrative support and coordination of Hybrid *ez*Parent
- **RAPP evaluator**: RAPP data manager and evaluator
- **RAPP leadership team**: community collaborative board providing leadership and input to RAPP programming
- **RAPP site**: community partner or organization providing *ez*Parent to families

**Table 1:** Description of parent program adaptations using FRAME^a^.

Modification		Goal of modification	Who was involved in the decision and modification?	Level of delivery^b^	Nature and goal of modification
**Contextual format** (**delivery modifications [eg, format, setting, personnel, and population])**					
	Videoconferenced group sessions	Intervention engagement and social connection	CPP^c^ or *ez*Parent, developers, RAPP^d^ coordinators, RAPP leadership, and team	Parent participants and facilitators	Parents complete the *ez*Parent independently and participate in 4 videoconferenced group sessions to promote *ez*Parent program completion and review of parenting strategies
	Groups facilitated by 1 CPP-trained group leader	Feasibility and cost	RAPP coordinators, RAPP leadership, and team	Facilitators	Smaller groups and less administrative burden for the group leader than in-person groups
	In-between group texts	Intervention engagement and social connection	CPP or *ez*Parent, developers, RAPP coordinators, and facilitators	Parent, participants, and facilitators	Text groups managed by the facilitators for weekly encouragement for program completion
	Provided tablets and hotspots to parents	Reach, engagement, and equitable access to all program components	RAPP coordinators, RAPP leadership, and team	Parent and participants	Parents receive tablets and short-term internet access to access *ez*Parent and participate in videoconferenced groups
**Training and evaluation (how staff are trained and how the intervention is evaluated)**					
	Facilitator training	Assure competency in *ez*Parent delivery and review virtual facilitator guide	CPP or *ez*Parent, developers, and RAPP coordinators	Facilitators	2-hour training to review *ez*Parent and the virtual session facilitator guide
	Fidelity assessment	Evaluate facilitator adherence to group session protocol and parent engagement	CPP or *ez*Parent, developers, and RAPP coordinators	CPP or *ez*Parent developers, RAPP coordinators, and facilitators	Developed a facilitator self-report fidelity assessment tool (adaptation of CPP fidelity checklist [23] to evaluate adherence to the session components)
**Implementation and scale up activities (strategies used to implement or spread the intervention)**					
	Administrative dashboard	Track *ez*Parent completion for stipend remittance to parents.	RAPP coordinators and CPP or *ez*Parent developers	RAPP coordinators, facilitators, CPP or *ez*Parent, and developers	A web-based administrative dashboard was created for RAPP coordinators and facilitators to track parent completion of ezParent modules
	Tech support	Support program delivery	RAPP coordinators, CPP or *ez*Parent, and developers	RAPP coordinators, CPP or *ez*Parent, and developers	Tech support link provided to parents for support with *ez*Parent program

^a^FRAME: Framework for Reporting Adaptations and Modifications–Expanded.

^b^Level of delivery=for whom or what is the modification made.

^c^CPP: Chicago Parent Program.

^d^RAPP: Rochester Area Parenting Program.

### Setting and Sample

The RAPP partnered with 6 community organizations (hereafter referred to as RAPP sites). These sites included a large Head Start and Early Head Start program serving over 1000 families; a child care center serving families of children aged 0 to 5 years; a community organization providing childcare, free education and comprehensive services to support Latino/a families; a community organization with the goal of optimizing an nurturing and supportive environment to empower women; a child care network providing early care and education services, including universal PreK; and the Rochester City School District Early Childhood Education Department, serving over 2800 children (aged 3-4 years) and their families.

### Parent Recruitment and Enrollment

The RAPP site managed parent recruitment to participate in the hybrid *ez*Parent and used successful recruitment techniques used for other programming for the parents they serve. Promotional flyers were provided by RAPP coordinators and emails were sent to parents to provide information about the *ez*Parent, provision of an android tablet, monetary incentives, and details on how to sign up. If there was in-person contact at the agency or school (eg, child drop-off or pickup), parents were provided with information regarding the program. All sites had a capacity of 10 parents per group. If more than 10 parents expressed interest, the sites kept a waiting list. In subsequent groups at the RAPP site, parents on the waitlist were invited first to participate.

Once the parents enrolled, they were invited to complete presurveys (refer to the Measures section) and received an android tablet computer that had been preloaded with the *ez*Parent program, zoom access, and internet access for the duration of program participation. Parents kept their android tablet at the end of the program for their personal use. Parents were instructed that all surveys throughout the course of the intervention were voluntary. A waiver of consent was obtained for these data as the parent data were anonymous and aggregated. In addition, parents were given instructions related to their first videoconferenced introductory group and paper copies of the curriculum handouts. Parents received US $20 for each group session and US $20 for completion of each module of *ez*Parent (ie, total possible US $200). These conditional cash incentives were provided using a debit card registered in the parents’ name. Conditional cash incentives have been shown to be an effective method for supporting engagement in programming [24,25]. The debit card was either sent to their address or hand delivered. Of note, using debit cards was selected by parents and the RAPP leadership team as the best and most effective way to compensate parents.

### Measures

#### RE-AIM Components

Table 2 provides a description of the RE-AIM components and corresponding variable and measure and data source for the implementation evaluation [21]. Study results will be presented by RE-AIM component and include facilitators and barriers to implementation.

**Table 2:** Variables and measures aligned with the RE-AIM^a^ component.

RE-AIM component and variable		Measure or data source
**Reach**		
	Number of groups that could be supported by agencies and parent participation	Administrative data^b^
	Reasons why parents choose to participate in the program	Presurvey
	Characteristics of the parent	Presurvey
	Parent program completion	*ez*Parent digital analytics and group attendance logs
**Efficacy**		
	Change in parent behavior	End-of-program survey
	Change in child behavior	End-of-program survey
	Parents’ satisfaction with the program	End-of-program survey
**Adoption**		
	Proportion of trained CPP^c^ group leaders adopting *ez*Parent hybrid	Administrative data
	Facilitators’ characteristics	CPP pretraining survey
**Implementation**		
	Facilitators’ adherence to group session protocol	*ez*Parent fidelity checklists
	Length of groups	*ez*Parent fidelity checklists and facilitator interviews
	Facilitators’ perception of group delivery	Facilitator interviews
	Implementation adaptations	Administrative data and facilitator interviews
	Implementation technical issues	Administrative data

^a^RE-AIM: Reach, Efficacy, Adoption, Implementation, and Maintenance.

^b^Administrative data are information collected by RAPP coordinators as part of ongoing management of program implementation.

^c^CPP: Chicago Parent Program.

#### Presurvey

Parents completed the presurvey before the first group session. The presurvey includes general parent demographics (ie, race and ethnicity, parent education level, and relationship with child) and an 11-item list of reasons for participating in the parenting program [26]. Parents selected all that applied to the list of reasons for participating with *no, yes,* and *most important*. Sample items include “I would like the chance to talk with other parents with young children,” “I would like help disciplining my child,” “I’m always looking for ways to be a better parent,” “I would like extra money for participating in the program,” and “I am required/have been asked to take a parenting class.”

#### Parent Program Completion

Digital tracking of use of the *ez*Parent program provides a measure of module completion. At the end of each *ez*Parent module, parents are awarded a module completion badge. Module completion is determined by parent receipt of the module completion badge. Facilitators and RAPP coordinators can view module completion in the administrative dashboard (Table 1) in real time. Group attendance logs are completed by the facilitators and submitted to RAPP coordinators for attendance tracking.

#### End-of-Program Survey

Parents were invited to complete an end-of-program survey after the last group session (approximately 7 weeks after baseline). This survey is an adaptation of the CPP and *ez*Parent end-of-program surveys [23,27]. The survey included items related to parent perception of the usefulness of the program in managing child’s behavior (2 items), perceived program impact on participant as a parent (2 items), overall program satisfaction (2 items), and acceptability of intervention delivery format procedures (3 items). In addition, parents responded to 3 open-ended prompts: What would you tell other parents who are interested in joining *ez*Parent? What did you find the most helpful or useful about the *ez*Parent Program for changing child behavior? What did you find the least helpful or useful in the *ez*Parent Program for changing child behavior?

### Characteristics of Facilitators

Facilitators participated in a 2-day CPP group leader training between 2018 and 2021. At the time of training, trainees completed a demographic survey that included their report of race, ethnicity, education, employment status, current position, and experience working with families of young children and leading groups with families. Either facilitators were paid a stipend or they facilitated the *ez*Parent groups as part of their work responsibilities.

### Hybrid ezParent Fidelity Checklists

After each videoconferenced group session, facilitators were invited to assess their adherence to the session protocol using a 7-item (session dependent) measure adapted from the CPP fidelity checklist [28]. Facilitators were not required to complete fidelity forms as the RAPP leadership team were concerned about burden on facilitators. Facilitators indicated *yes* or *no,* depending on whether they performed the expected action during the group session. Examples of adherence items include review of group ground rules, review of module summary of important points, and discussion of the *ez*Parent module practice assignment. In addition, facilitators self-reported the length of time of the group (in minutes), rated parents level of engagement (eg, the overall groups interest and active involvement in the discussion) during the group session on a 3-point scale (ie*, high*, *mixed*, *and low*), and assessed the extent parents seemed to understand the program strategies (ie, *high*, *moderate*, *and low*). Finally, facilitators could provide additional comments or feedback as an open-ended response.

### Administrative Data

As part of program implementation, the RAPP coordinators monitored the number of groups that could be supported by RAPP sites and parent participation. This was driven by funding constraints, and coordinators balanced program costs to increase the number of groups available to reach the maximum number of parents. Parents using the *ez*Parent program could report technical issues related to *ez*Parent through a link in the program or contact coordinators for other technical support needs. Data logs of technical issues were maintained by the coordinators. Finally, coordinators met with the facilitators on an as needed basis to meet specific training and implementation needs. Coordinators kept a log of any implementation adaptations reported during these meetings.

### RAPP Facilitator Interviews

In spring 2022, 8 facilitators who were currently involved with RAPP and hybrid *ez*Parent delivery at the time of the evaluation were invited to participate in a brief (<30 minutes) postprogram interview. The goal of the interview was to assess facilitators opinions regarding the hybrid delivery methods (ie, implementation in RE-AIM framework), use of the discussion guide to support the conduct of the group session, and evaluate the strengths and weaknesses of the implementation model. An interview guide was developed (Multimedia Appendix 1) and interviews were conducted by 2 of the authors (RP and SMB) who were independent of the RAPP. Interviews were not recorded; detailed notes were taken during the interview. In total, 6 participants (response rate: 6/8, 75%) completed the informed consent process and the phone interview. Participants received a US $25 gift card as a thank you for their time and input.

### Data Analysis

All statistical analyses were conducted using SPSS (version 29; IBM Corp) [29]. Descriptive statistics were used to describe the sample and the reach, adoption, and implementation components of the RE-AIM model.

A thematic analysis focused on identifying implementation outcomes aligned with the implementation component of the RE-AIM model. Specific implementation categories were identified a priori (ie, facilitator adherence to group session protocol, length of group sessions, and facilitator perception of group delivery and parent engagement). Two authors (RP and SMB) reviewed the data independently, then met to review quotes for consensus and alignment with the implementation variables. Data from the interviews were used in a convergent design [30] to corroborate and provide examples of the quantitative data.

## Results

### Reach

#### Number of Groups and Parent Motivation for Participation

From September 1, 2020, to August 1, 2022, a total of 24 hybrid *ez*Parent groups were conducted by 13 facilitators. The goal for each group was 10 parents per group and the average group size was 10 (SD 0.42). All enrolled parents (n=240) were invited but not required to complete the pre- and postsurveys. In total, 77.9% (187/240) of eligible parents responded to the presurvey and 61.3% (147/240) of the eligible parents responded to the postsurvey. Of the 187 parents responding to the presurvey, 180 (96.3%) endorsed at least 1 motivation for participating in the program. Parents could endorse multiple motivations as *most important*. As shown in Table 3, the top-rated items endorsed as a motivator by over 94% of the respondents included “I would like to learn better ways to communicate with my child,” “I’m always looking for ways to be a better parent,” and “I would like to learn better ways of managing my child’s behavior.” The top items not endorsed as *most important* included “I am required/have been asked to take a parenting class” and “Another parent recommended I take this program.”

**Table 3:** Reasons parents (n=180) endorsed motivating their participation in the program^a^.

Motivation	Most important, n (%)	Yes, n (%)	No, n (%)
I’m always looking for ways to be a better parent^b^.	101 (56.4)	73 (40.8)	5 (2.8)
I would like to learn better ways to communicate with my child.	91 (50.6)	85 (47.2)	4 (2.2)
I would like to learn better ways of managing my child’s behavior.	82 (45.6)	88 (48.9)	10 (5.6)
I would like the chance to talk with other parents with young children.	33 (18.3)	128 (71.1)	19 (10.6)
I would like help disciplining my child^b^.	30 (16.8)	84 (46.9)	65 (36.3)
I was motivated by the recruiter to take this program^b^.	18 (10.1)	88 (49.2)	73 (40.8)
I would like the extra money for using the new parenting skills with my child.	15 (8.3)	104 (57.8)	61 (33.9)
I would like the extra money for attending the parent group.	8 (4.4)	109 (60.6)	63 (35)
I am required/have been asked to take a parenting class.	6 (3.3)	12 (6.7)	162 (90)
My child’s teacher recommended that I participate in this program.	3 (1.7)	55 (30.6)	122 (67.8)
Another parent recommended I take this program^b^.	3 (1.7)	43 (24.0)	133 (74.3)

^a^Parents could select multiple items as most important.

^b^One respondent picked multiple conflicting responses (eg, “no” and “most important”); therefore, these were not included in the summary.

#### Parent Characteristics

Of the 187 parents responding to the presurvey, 112 (59.9%) identified as Black or African American, 46 (24.6%) as White, and 42 (22.5%) identified as Hispanic. Of the 103 parents who reported their relationship with their child, 90 (87.4%) were mothers, 8 (7.8%) were fathers or stepfathers, and 5 (4.9%) were foster parents or grandmothers. Finally, parents (n=180) reported their highest level of education being less than high school (n=16, 8.9%), high school (n=59, 32.8%), some college (no degree; n=46, 25.6%), associate degree (n=29, 16.1%), bachelor’s degree (n=15, 8.3%), and graduate degree (n=15, 8.3%).

#### Parent Program Completion

On average, parents (n=240) completed 4.58 (SD 2.43) of the 6 *ez*Parent modules and 72.1% (173/240) of the parents completed all 6 modules. A total of 47 (19.6%) parents did not complete a single module. Average group attendance across all participants (N=240) was 71.2% across the 4 sessions. Of those who attended at least 1 group session (219/240, 91.3%), average attendance was 77.7% across the 4 sessions.

#### Parent Satisfaction and Parent and Child Behavior

Parents reported high levels of satisfaction with the program and improvements in their feelings of self-efficacy and their child’s behavior following their participation in *ez*Parent hybrid. Specifically, 81.5% (110/135) reported that the concerns they had about their child’s behavior were *better* or *much better* and 82.6% (109/132) felt their child’s behavior was *better* or *much better* following their participation in hybrid *ez*Parent. Before participating in hybrid *ez*Parent, 42.5% (57/134) of the parents indicated that they were *not at all* (n=13) or *a little* (n=44) confident about managing their child’s behavior and 57.5% (77/134) reported feeling *confident* (n=45) or *very confident* (n=32) managing their child’s behavior. After participating in hybrid *ez*Parent, 92% (126/137) of these same parents reported they were *confident* (51/137, 37.2%) or *very confident* (75/137, 54.7%) about managing their child’s behavior, while 8% (11/137) remained *not at all* or *a little* confident about managing their child’s behavior.

#### Parent Satisfaction

Parents (n=137) responded to a program satisfaction question and 89% reported they were very satisfied (n=90, 65.7%) or satisfied (n=32, 23.4%) and 10.9% (n=15) of parents reported they were either dissatisfied (n=10, 7%) or very dissatisfied (n=6, 4%) with the program. When asked if they would recommend hybrid *ez*Parent to another parent, responding parents (n=117) reported they would highly recommend (n=94, 80.3%) or recommend (n=21, 17.9%) the program.

Most parents (104/117, 88.9%) said it was not at all hard to use the *ez*Parent program and 83.7% (98/117) felt the time spent using the program was “just right.” Similarly, 88% (103/117) of the parents found the *ez*Parent program very helpful and 91.2% (107/117) found completing the module practice assignments was “not at all hard.” Although these group sessions were conducted remotely, 71.8% (84/117) of the parents indicated their intention to remain in contact with other members of their group.

### Adoption

Of the 23 facilitators who conducted in-person CPP groups sessions, 11 (48%) facilitated *ez*Parent group sessions. Two additional trained facilitators ran *ez*Parent sessions who had not previously conducted in-person groups. Overall, this was a diverse group of facilitators with 69% (9/13) identifying as Black, 17% (2/12) identifying as White, and 17% (2/12) identifying as American Indian (individuals could select all that apply). In addition, 17% (2/12) of the facilitators identified as Hispanic ethnicity. Of the 13 *ez*Parent facilitators, 2 (15%) reported having a high school degree, 5 (39%) having an associate degree or some college, with the remaining of the sample having a bachelor’s degree (n=2, 15%) or a graduate degree (n=4, 31%). Facilitator (N=13) experience at the time of training varied with greater experience working with individual families (*a lot*: n=6, 46%; *quite a bit/some*: n=7, 54%) and less experience leading groups of families *a lot*; n=2, 15%; *quite a bit/some*; n=10, 77%; *a little*; n=1, 8%).

### Implementation

In total, 38% (n=5) of the 13 facilitators completed end of session adherence self-reports, representing 31 of the 96 group sessions (32.3%). In addition, 6 of the 8 current facilitators participated in the facilitator interviews. Refer to Table 4 for facilitator quantitative and qualitative themes for implementation from adherence self-reports and facilitator interviews. Overall, facilitators found the hybrid delivery easy to implement and reported high parent engagement and understanding of CPP strategies. Implementation technical issues were infrequent and related to issues with the parent tablet which were administratively resolved. Only 1 technical issue was reported for the *ez*Parent program and was related to logging in. RAPP coordinators experienced technical issues related to the *ez*Parent administrative dashboard that was developed for this project. Because of these technical issues, *ez*Parent prepared monthly parent use reports for the RAPP coordinators to assure accurate use data for parent incentives.

Times of the group meetings were determined based on parent polls, most groups occurred on weekday evenings (ie, Monday through Thursday) between 5 PM and 7 PM, 1 group was held at lunch time (ie, noon to 1 PM) and 1 group was held on a Friday evening from 6 PM to 7 PM. No parents indicated wanting a weekend meeting time. Facilitators reported several adaptations to address challenges to hybrid delivery.

Three main challenges were identified by facilitators, including completing a group session in 1 hour, fostering parent engagement in the program, and building connection in the virtual environment. As 1 facilitator noted, “for in-person groups we had more bonding time, it was hard to build relationships virtually.” To address these challenges, several facilitators would stay on the videoconference an extra 30 minutes to provide time for the parents to continue sharing. A facilitator reported, “I would say I’m here to stay on and many would stay on to discuss, talk it out, vent, I let them. They need that. In person they would have the time they needed.” In addition, facilitators created text groups and provided text reminders for group sessions and to engage parents in completing the practice assignment with their children. As 1 facilitator noted “one thing I liked was the text messaging—once I started doing that—parents would thank me for reminding them—they liked that*.*”

Although the facilitators felt positive about the group session delivery, they reported needing to tailor facilitation methods for videoconferenced groups. This included creating strategies to keep parents’ attention and promoting group sharing, as one facilitator noted, “I had to get creative to keep everyone’s attention.” Strategies included, asking parents to keep videos on, calling parents by name, and responding to nonverbal behaviors. One facilitator reported “For the last session I did a round robin and asked each parent what their take was away from completing the modules. I also asked if they were practicing. Each parent shared what really stood out for them and what helped them the most*.*”

Despite the challenges, facilitators found the hybrid delivery convenient for them and for parents. Facilitators reported that the hybrid delivery was logistically easier for them compared with in-person groups. In addition, although many reported that there was some loss in group connection compared with in-person groups, facilitators agreed that the convenience and ability for parents to participate in the “comfort of their own homes” might outweigh the challenges. Additional benefits of delivering the hybrid program included managing childcare (for both parents and facilitators), not having to spend time in gathering food and beverages for in-person group meetings, and not needing to spend time driving to and from the group. In addition, facilitators reported that parents were highly engaged and would readily report what they were learning from the program and changing in their own lives and the group session helped parents learn from each other’s experiences.

**Table 4:** Implementation outcomes.

Implementation variable	Quantitative (RAPP^a^ facilitator self-report; n=31 group sessions)	Qualitative (RAPP facilitator interviews; n=6)^b^
Facilitator adherence to group sessions	92% facilitator adherence to the group protocols^b^ 57% (17/30) *agree^c^ or strongly agree* that the guide was helpful in running group sessions	Facilitators reported high consistency in delivery and the group session guide was helpful. “Every time I start a new class—go back to the group book—and I get how to facilitate the session” “Followed the facilitator guide closely; it was really helpful” “Used the script as a guide—but not so stringent. Always feel like room for flexibility...”
Length of groups	Mean 60.5 (SD 14.7; range 34-90) minute 73% (22/30) reported *about the right amount of time* and 27% (8/30) reported *not enough time for group discussion*.	Facilitators reported parents wanted to talk more and sessions often would run over time. “...a little more time is always needed. One hour is not enough time to cover and allow everyone to be able to express what they are feeling, experiencing and get the feedback needed to help motivate and encourage.” “I felt like I am rushing the parents through the conversations” “One and half hour would be ideal. There were plenty of times that conversation could continue” “I think if we stay virtual, not a bad thing—need to add more time or do weekly”
Facilitator perception of group delivery and parent engagement	Parent engagement was *high* (23/31, 74%) and *mixed* (8/31, 26%). Parent understanding of program strategies was *high* (18/22, 82%) and *moderate* (4/22, 18%). Affective tone of the group was *positive* (30/31, 97%) and *neutral* (1/31, 3%).	Parents came to the groups consistently and web-based delivery was convenient for them. “Felt like got better attendance in virtual than in person” “I think it is a very safe method—families feel comfortable” “Parents in the group reviewed everything so they would know what was coming” Parents were overall engaged but often multitasked during the sessions. “I could tell they were engaged because of the conversation—it would be consistent” “Would often see parents nodding, paying attention, a matter of everyone joining in and they would share” “See them cooking dinner, feeding children” “They have the kids in and out—trying to do the session and getting interrupted” The group discussions reflected grasp of material learned from the ezParent module and parents discussed putting the parent strategies learned in the modules into practice in their household. “Several parents would say we tried this, most of them had an example of what worked” “One father even shared that he now dances with his son—something that he never did before.” “There was one parent who said that all of them helped her because she is learning how to take care of herself so that she can take better care of her children.” “One parent told the group that the child now has a schedule that she even follows on the weekend.” “I was skeptical, but the program does work.”

^a^RAPP: Rochester Area Parenting Program.

^b^Adherence frequency is based on a sum the adherence items (session-dependent) across the 31 fidelity reports received from the group leaders.

^c^Italicized words represent survey response choices.

## Discussion

### Principal Findings

The purpose of this paper was to describe the implementation outcomes of the hybrid delivery model of *ez*Parent during community-based dissemination in response to the COVID-19 pandemic lockdown causing in-person intervention to be unavailable. Using the RE-AIM framework, we found that the *ez*Parent program was successful in reaching a diverse sample from the city of Rochester, New York, resulting in high levels of parent satisfaction and improvements in confidence in managing their child’s behaviors. Our findings for parent satisfaction are consistent with prior research investigating general parent satisfaction with *ez*Parent [31]. The improvements in parental confidence in managing their child’s behaviors within hybrid *ez*Parent is consistent with prior research conducted with CPP [13].

Overall, the trained facilitators easily adopted the new delivery format and implemented the sessions with minimal difficulties. As the aim of launching hybrid *ez*Parent was not to conduct research but support the community during the COVID-19 pandemic, we were unable to determine potential sustainability (ie, *M*aintenance) as proposed by the RE-AIM framework. While we were able to demonstrate success within the implementation of *ez*Parent, it is imperative to identify facilitators and barriers to implementation delivery to support the sustainability of the implementation of the program using the hybrid delivery model.

### Facilitators and Barriers to Implementation

Factors that eased *ez*Parent implementation included the support and strength of the RAPP leadership team relating to decisive actions in implementing the new modality, the use of community-based facilitators who were familiar with the community which was represented in the group and in CPP delivery, flexibility of timing of the group sessions including the length of the meeting (60-90 minutes), parental preference in day and time of meetings, and ability to participate from anywhere. Group day and times were parent driven, not chosen by the facilitator or the community partner. One facilitator polled parents for the best time to meet and was overwhelmed and challenged by the variety of responses. We recommend community organizations provide parents with a few meeting times to lessen variability while providing an opportunity for parents to choose. Most groups were held between 5 PM and 7 PM on weekday evenings as parents identified this as the best timeframe. However, there were competing priorities during synchronous group sessions, including childcare or preparing dinner that may affect parent engagement. However, many parents were able to creatively address these competing priorities. For example, some parents arranged their meal break at work for the group session. We believe this flexibility contributed to the proportion of parents who attended at least one session (91.3%) and of those who engaged in all 4 group sessions (77.7%). Attendance in this sample was slightly below parent attendance rates recently reported for videoconferenced delivery of 12-session CPP (82.3% of the enrolled parents attended at least 1 group session) [32]. We calculated our engagement metrics (ezParent use and group attendance) for all parents who enrolled in the program regardless of attending group to assess program adoption.

In addition to providing access and flexibility for parent engagement, the RAPP facilitators enjoyed the flexibility in the internet-based groups and conserving efforts and resources to manage in-person meetings. However, increased flexibility seemed to come with a loss of the reported social connection that came with in-person groups. Therefore, facilitators used other tools, such as group text messaging to stay in contact with parents between group sessions and to help build and maintain a sense of community. Social connection and building community are vital components to successful group interventions and the pandemic gave rise to challenges in maintaining social connectedness. The facilitators’ use of digital tools to create, maintain, and enhance a sense of community within their groups were found to be useful ways to engage parents and create community. These results are consistent with Plesko et al [32], highlighting the challenges with moving from in-person CPP to web-based group meetings.

We recommend using a variety of communication means to engage parents in-between sessions to support completion of the *ez*Parent module, support parent enactment and practice of parenting skills, and to remind parents of the next scheduled group sessions. Although not included in our hybrid delivery model, we suggest including a booster session 6 to 8 weeks after the last live session to determine if parents have any questions or barriers to practicing and implementing new behaviors to support their children. This will also aid in obtaining data for the Maintenance component of the RE-AIM framework.

### Limitations and Strengths of the Project

#### Evaluation Metrics

When developing the hybrid delivery model, priorities were focused on the community needs during the height of a global pandemic and not on the project’s evaluation. Upon analysis of the outcomes, we determined that our measures were not set up in a way that supported investigating parent changes over time or evaluating baseline data relationship with program engagement. For example, using unique identifiers for the baseline and follow-up questionnaires is planned for RAPP delivery of hybrid *ez*Parent starting August 2024. In our evaluation, the lack of paired surveys allowed only for independent and unrelated group analyses; however, in conducting the evaluation, we were able to identify this need highlighting the importance of academic and community-based organization relationships. The strengths that each partner brings to hybrid *ez*Parent will allow for continued support in reaching the most underserved populations in the Rochester area and an opportunity for more formal and robust outcome evaluations in the future. An additional strength includes the inclusion of facilitator interviews to examine perceptions of implementation outcomes. This provides a first-hand account of the benefits and drawbacks of the videoconferenced group sessions and examples of problem-solving that facilitators implemented to best support parents’ needs.

#### Parental Motivation in Participating

Parent motivation is an important factor in PT delivery; thus, a question was asked about the parent’s motivation in participating in hybrid *ez*Parent. However, respondents could select all that apply for motivational reasoning, and we could not determine each parent’s primary motivation to participate. Given the nature of the responses, it was difficult to determine the most salient reason for participating; however, our results showing parents endorsing, “I’m always looking for ways to be a better parent” as a motivator is consistent with prior research [25]. In addition, most selected items for motivation related to strengthening parenting skills and supporting their child. For evaluation purposes, we recommend implementing a primary motivation question and then a way to assess other motivations to see if results differ by motivation factor.

Although approximately 8% (15/180) of the parents identified the financial incentive as a *most important* motivator for participating, 57.8% (104/180) to 60.6% (109/180) endorsed incentives as *a* motivator to attend sessions and complete practice. This finding is consistent with previous work related to incentive-based attendance as motivators for behavioral change interventions [24,25]. In a previous study of CPP and financial incentives, 71% of parents identified receiving extra money as a motivator and this was a predictor of program attendance [25]. In this study, the *ez*Parent dissemination was part of a community-led initiative through RAPP and funded through these programs, representing a sustainable method for maintaining implementation and supporting program engagement. Although providing financial incentives and a tablet for program access increases the overall cost of program delivery, these barrier reduction strategies may be important to support program access and parent engagement.

### Lessons Learned

While reflecting on the implementation and evaluation of hybrid *ez*Parent, there are several lessons learned for future community-based dissemination. During year 1 of implementation, parents were sent a web-based link for the presurvey and anecdotal comments, which indicated that this was seen as impersonal and not particularly relevant for parents to complete. In year 2, we changed this to a paper survey being administered at the time of disbursement of the tablet. This change resulted in a more robust completion of the presurvey by parents. In addition, parents were not incentivized to complete the presurvey and postsurvey; therefore, we suggest that parent participants receive an incentive to complete surveys. This may improve response rates and allow for more rigorous evaluation of the program outcomes.

An *ez*Parent administrative dashboard was developed for this project; however, RAPP coordinators reported multiple technical issues. On the basis of these concerns, revisions have been made to the dashboard and we anticipate more autonomy for RAPP coordinators to process desired reports accurately and efficiently. In addition, further refinements of the *ez*Parent dashboard have been implemented based on these findings and others [33] to assure relevance to community organizations implementing *ez*Parent. For example, the ezParent dashboard now allows lists of users to be created based on cohort as well as the main page having a summary of users. If the user needs more detailed information on a participant, they can easily access the information from the main page.

Our results indicate that using the hybrid *ez*Parent is a feasible and effective way to engage parents. We also found that facilitators and parents enjoyed the flexibility that a web-based group session provides. Future research should comprise of more rigorous evaluation processes so that changes in the outcomes of hybrid *ez*Parent participants can be explored more precisely. In addition, more research needs to be conducted on the processes surrounding implementation of the *ez*Parent program and its dissemination.

## Appendix

Multimedia Appendix 1 Interview script.

## Abbreviations

CPP: Chicago Parent Program
FRAME: Framework for Reporting Adaptations and Modifications–Extended
PT: parent training
RAPP: Rochester Area Parenting Program
RE-AIM: Reach, Efficacy, Adoption, Implementation, and Maintenance
