# Supplementary material for: Implementation of a Parent Training Program During Community-Based Dissemination (From In-Person to Hybrid): Mixed Methods Evaluation
Source: JMIR Pediatr Parent. 2024 Jul 3;7:e55280. doi: 10.2196/55280 (PMC11255538; doi:10.2196/55280)
Supplement: Multimedia Appendix 1 [file pediatrics_v7i1e55280_app1.docx]

**Interview Guide for Facilitator Interviews**

1. What were your impressions of the *ez*Parent facilitator discussion guide?

Prompts:

- 1. Do you think there were any components in the discussion guide that were unclear? Describe.

1. How did you use the discussion guide and how did it help you prepare for conducting the virtual sessions?

Prompts:

- 1. Do you feel having the discussion guide was beneficial for you?
  2. Is there something other than the discussion guide that you think would be useful for facilitators?

1. Tell me about parent engagement in the group sessions?

Prompts:

- 1. How did parents show engagement in group sessions?
  2. Did the parents share their experience using the interventions learned in the modules? Give examples.

1. In your opinion, is one hour enough time for the virtual discussion groups?

Prompts:

- 1. If not, what would you change? Would you remove certain components or extend the meeting time?
  2. Did you feel like you had to rush through certain components or skip out on anything?

1. In thinking about the virtual groups, What worked well? What were challenges? What would you change?
2. In your opinion, did you feel like the virtual groups were useful?
   1. What did virtual groups add to the *ez*Parent program?
   2. Would you prefer a different delivery format?
